# Crystal structure of a raw-starch-degrading bacterial α-amylase belonging to subfamily 37 of the glycoside hydrolase family GH13

**DOI:** 10.1038/srep44067

**Published:** 2017-03-17

**Authors:** Yanhong Liu, Jigang Yu, Fudong Li, Hui Peng, Xuecheng Zhang, Yazhong Xiao, Chao He

**Affiliations:** 1 Anhui Provincial Engineering Technology Research Center of Microorganisms and Biocatalysis and School of Life Sciences, Anhui University, Hefei, Anhui 230601, China; 2 Hefei National Laboratory for Physical Sciences at Microscale and School of Life Sciences, University of Science and Technology of China, Hefei, Anhui 230026, China

## Abstract

Subfamily 37 of the glycoside hydrolase family GH13 was recently established on the basis of the discovery of a novel α-amylase, designated AmyP, from a marine metagenomic library. AmyP exhibits raw-starch-degrading activity and consists of an N-terminal catalytic domain and a C-terminal starch-binding domain. To understand this newest subfamily, we determined the crystal structure of the catalytic domain of AmyP, named AmyP_ΔSBD_, complexed with maltose, and the crystal structure of the E221Q mutant AmyP_ΔSBD_ complexed with maltotriose. Glu221 is one of the three conserved catalytic residues, and AmyP is inactivated by the E221Q mutation. Domain B of AmyP_ΔSBD_ forms a loop that protrudes from domain A, stabilizes the conformation of the active site and increases the thermostability of the enzyme. A new calcium ion is situated adjacent to the -3 subsite binding loop and may be responsible for the increased thermostability of the enzyme after the addition of calcium. Moreover, Tyr36 participates in both stacking and hydrogen bonding interactions with the sugar motif at subsite -3. This work provides the first insights into the structure of α-amylases belonging to subfamily 37 of GH13 and may contribute to the rational design of α-amylase mutants with enhanced performance in biotechnological applications.

α-Amylases (EC 3.2.1.1) are endo-hydrolases that act on α-(1,4)-glucosidic linkages in starch and other related oligo- and polysaccharides, thus causing the release of malto-oligosaccharides and glucose in the α-anomeric form. α-Amylases are essential for the conversion of starch into oligosaccharides and are critical for many organisms that use starch as a primary source of energy. α-Amylases also have potential applications in a number of industrial processes, such as processes in the food, fermentation, and pharmaceutical industries.

According to the Carbohydrate-Active enZymes (CAZy) database, glycoside hydrolase family 13 (GH13) is the main α-amylase family that, which, together with GH70 and GH77, forms clan GH-H, in which the catalytic domain adopts a (β/α)_8_-barrel. GH13 α-amylases use a reaction mechanism that retains anomeric configuration, and share 4–7 conserved sequence regions and catalytic machinery^1^. The observation that some GH13 members exhibit high mutual sequence similarities has led to the current division of the GH13 family into 42 subfamilies. The GH13_37 subfamily is the most recently established α-amylase subfamily^2^. It was created on the basis of the isolation and phylogenetic analysis of a novel α-amylase, designated AmyP, from a marine metagenomic library. AmyP shares 85.6% sequence identity with the glycosidase A0A0B9G5J0 from the bacterium *Photobacterium gaetbulicola*. AmyP is 639 amino acids in length and has a theoretical molecular weight of approximately 70 kD. It has been proposed to contain a conserved catalytic domain at the N terminus^2^ and a starch binding domain at the C terminus^3^. AmyP exhibits high substrate specificity for starch over pullulan or cyclomaltodextrins, efficiently hydrolyzing starch into glucose, maltose and maltotriose^2^. Currently, the GH13_37 subfamily contains, in addition to AmyP, approximately 56 hypothetical members, most of which are found in marine bacteria.

In general, GH13 α-amylases are three-domain proteins, consisting of domains A, B and C. Of special interest is that subfamily GH13_37 members may lack domain B because the loop connecting strand β3 to helix α3 of their catalytic (β/α)_8_-barrel appears to be too short to form a B domain typical of the GH13 family. For several α-amylases, it has been reported that the metal and substrate binding sites are located in domain B and its interface with domain A and that these regions affect the activity and stability of the enzyme^4^,^5^,^6^,^7^,^8^. The specific role of domain B in the GH13_37 subfamily will be clearer when the three-dimensional structure of the novel α-amylase AmyP has been solved and described in detail.

AmyP is a raw-starch-degrading enzyme (RSDE), thus making it of potential interest in the food processing and green-fuels communities. AmyP exhibits the unique and remarkable ability to preferentially and very rapidly digest raw rice starch. The specific activity of AmyP at 40 °C against 4% raw rice starch reaches 118.5 ± 0.6 U/mg, which is much higher than its activity against other raw starches^9^. Maximal hydrolysis by AmyP occurs within 4 h for 10 mg/ml and within 1 h for 80 mg/ml raw rice starch, thus indicating a very rapid rate of hydrolysis.

Although much work on the production, purification, characterization, and application of microbial RSDEs has been conducted in recent years^10^, studies focusing on the mechanism of action of RSDEs are rare. Most bacterial and fungal RSDEs possess starch-binding domains (SBDs), usually at the C terminus, that mediate attachment to starch granules^11^,^12^ and in some cases disrupt the surface of the granules^13^. It has previously been shown that the C-terminal domain of AmyP has an affinity for raw rice starch granules, that is similar to that of full-length AmyP, whereas the binding of the catalytic domain of AmyP, designated AmyP_ΔSBD_, to raw starch granules is so weak that it is difficult to quantify. This result suggests that the C-terminal domain of AmyP can be unambiguously defined as an SBD and that the binding of AmyP to raw starch depends primarily on its SBD. However, some amylolytic enzymes that lack an SBD domain are capable of binding and digesting raw starch. These enzymes include the barley α-amylase isozyme AMY1^14^,^15^,^16^,^17^, yeast glucoamylase from *Saccharomycopsis fibuligera*^18^, and human salivary α-amylase^19^, which have well-defined structures and possess one or more secondary carbohydrate-binding sites called surface-binding sites (SBSs) on the catalytic domain or an intimately associated domain rather than on an SBD. The SBSs usually contain aromatic residues. Recently, the bacterial α-amylases from *Anoxybacillus* species (ASKA and ADTA)^20^, *Geobacillus thermoleovorans* (GTA^21^, Pizzo α-amylase^22^, and GtamyII^23^), and *Bacillus aquimaris* (BaqA^24^) have been proposed to constitute a novel GH13 subfamily^25^ of raw-starch-digesting α-amylases, that lack SBDs but exhibit the ability to degrade raw starch. However, enzymes in this subfamily possess an extended C-terminal region that is rich in aromatic residues, and the actual role of this region remains to be investigated. Likewise, AmyP_ΔSBD_ retains raw-starch digesting ability and has a specific activity of 55.5 ± 1.4 U/mg for 4% raw rice starch^3^. Additionally, another GH13_37 α-amylase that does not possess a distinct SBD, AmyASS from *Aeromonas salmonicida*, is able to degrade raw rice starch^26^. Thus, the knowledge of the three-dimensional structure of AmyP may improve understanding of the mechanism of action of RSDEs.

Here, we present the first crystal structures of α-amylase from the GH13_37 subfamily. This work provides further insights into the structure of α-amylases that can directly degrade raw starch granules.

## Results

### Overall structure

The catalytic residues of AmyP were initially identified by comparing the amino acid sequence of AmyP with sequences in the Protein Data Bank. Substitution of the glutamic acid at position 221 of AmyP with glutamine (E221Q mutation) resulted in nearly complete inactivation of the enzyme. This inactivation was confirmed by enzyme activity assays using 10 mg/ml of soluble starch as substrate. The E221Q mutant displayed 0.125% of the activity of the wild-type (WT) enzyme in thses assays. The crystal structure of the E221Q catalytic domain mutant AmyP_ΔSBD_ in complex with maltotriose was obtained through co-crystallization of the E221Q mutant of full-length AmyP with γ-cyclodextrin (γ-CD) (AmyP-E221Q/γ-CD). This structure was determined to a resolution of 2.55 Å by molecular replacement by using the crystal structure of the *Bacillus stearothermophilus* TRS40 neopullulanase (pdb accession code 1J0H^27^) as a search model. The crystal structure of WT AmyP_ΔSBD_ complexed with maltose was obtained through co-crystallization of full-length WT AmyP with β-cyclodextrin (β-CD), an unpreferred substrate of AmyP (AmyP/β-CD). This structure was determined to a resolution of 1.95 Å by using the AmyP-E221Q/γ-CD structure as an initial model.

Both crystal structures contain four enzyme molecules in the asymmetric unit. Surprisingly, each molecule includes residues 1 to 496, corresponding to AmyP_ΔSBD_, the theoretical molecular weight of approximately 55 kD. Nevertheless, no electron density is invisible for residues 497–639, which correspond to the C-terminal SBD domain. Peptide sequencing of protein bands from the dissolved crystal sample by sodium dodecyl sulfate polyacrylamide gel electrophoresis (SDS-PAGE) and liquid chromatography-mass spectrometry (LC-MS) showed that partial degradation of the SBD domain of the AmyP-E221Q/γ-CD crystals had occurred (Fig. S1a). The far-UV circular dichroism (CD) spectrum showed that the complete SBD domain has some secondary structure, and is not totally disordered (Fig. S1b). These results suggested that the SBD region of recombinant AmyP expressed in *Escherichia coli* cells might be poorly organized, thus resulting in its absence in the crystal structure.

**Figure 1:**
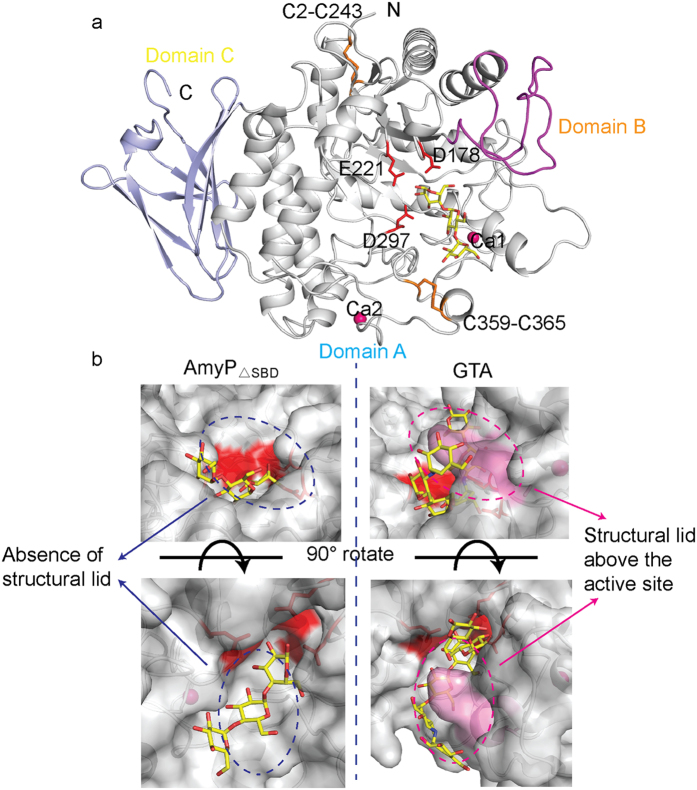
Overall structure of AmyP_ΔSBD_. (**a**) AmyP_ΔSBD_ consists of domains A (shown in gray), B(shown in magenta) and C(shown in blue). Disulfide bridges are shown in orange. Calcium ions are shown as hot pink spheres. Catalytic residues are shown as red sticks. (**b**) Structural comparison of active sites of AmyP_ΔSBD_ and GTA (pdb accession code 4e2o) shows the absence of a protruding lid above the active site for AmyP_ΔSBD_. The lid in GTA (shown in violet) is involved in the stabilization of the substrate. The catalytic residues are shown in red.

The AmyP_ΔSBD_ is composed of A, B, and C domains and shares structural features with other GH13 α-amylases (Fig. 1a). Domain A (residues 1–125 and 155–410) forms of a (β/α)_8_ barrel that contains the catalytic site. Domain B (residues 126–154) forms a loop that protrudes from domain A and has a non-regular secondary structure. Domain C (residues 411–498) consists of an antiparallel eight-stranded β-sheet. In addition, four cysteine residues in domain A are involved in two disulfide bridges (Cys2-Cys243 and Cys359-Cys365) that are conserved in the GH13_37 subfamily (Fig. S2). The locations of the disulfide bridges in AmyP_ΔSBD_ are not identical to the locations of the disulfide bridges in chloride-dependent α-amylases^28^,^29^.

A search on the Dali server revealed that AmyP_ΔSBD_ is structurally most similar to the *B. stearothermophilus* neopullulanase excluding domain N (pdb accession code 1J0H^27^, z-score 41.1 and rmsd of 2.0 Å for 279 Ca atoms, with 28% sequence identity), *G. thermoleovorans* α-amylase GTA lacking transmembrane regions (pdb accession code 4e2o^21^, z-score 39.6 and rmsd of 2.2 Å for 288 Ca atoms, with 26% sequence identity), and *Thermus* maltogenic amylase excluding domain N (pdb accession code 1GVI^30^, z-score 39.5 and rmsd of 2.2 Å for 288 Ca atoms, with 27% sequence identity). The most divergent portion of the structure lies within the B domain, which is adjacent to the active site. The B domain of AmyP_ΔSBD_ is indeed considerably shorter than the B domains of α-amylases belonging to other GH13 subfamilies. It is composed of only loop elements, does not bind metal ions and points away from the catalytic site. Structural comparison of AmyP_ΔSBD_ and GTA showed that the B domain loop of AmyP_ΔSBD_ lacks a region corresponding to residues 181–184 of GTA, which form a small structural lid above the active site (Fig. 1B). Owing to the small size of domain B in AmyP_ΔSBD_, a much wider and more open substrate binding pocket is created, which may facilitate access to starch granule surfaces and increase the rate of hydrolysis of raw starch.

### Ca^2+^ ion binding to AmyP_ΔSBD_

**Figure 2:**
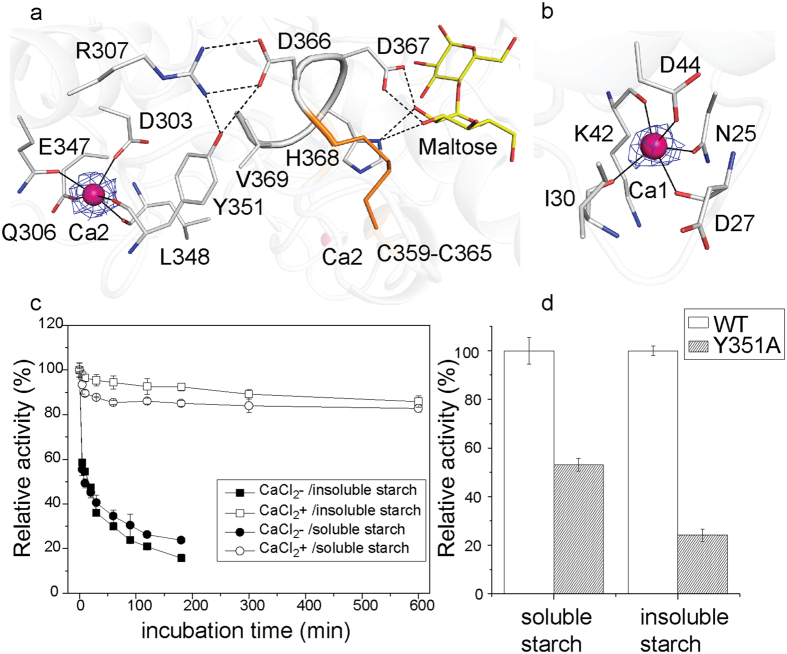
Calcium ion binding sites in AmyP_ΔSBD_. (**a**) Stick representation of the Ca2 binding site and the residues surrounding Ca2. Ca2 is coordinated by the main-chain oxygens of Leu348 and Tyr351 and the side-chain oxygens of Asp303, Gln306 and Glu347 in domain A. Close-up view showing the 2*Fo*–*Fc* electron density pattern (2σ contoured) of Ca2. Ca2 may help maintain the conformations of Arg307 and Tyr351, thereby stabilizing the substrate binding loop formed by Asp366-Val369. Hydrogen bonds are shown as dashed lines. (**b**) Stick representation of the Ca1 binding site. Ca1 is bound to the main-chain oxygens of Asp27, Ile30 and Lys42, and the side chain oxygens of Asn25 and Asp44 from domain A. Close-up view showing the 2*Fo*–*Fc* electron density pattern (2σ contoured) of Ca1. (**c**) Thermal inactivation of WT AmyP_ΔSBD_ incubated in the absence or in the presence of 10 mM CaCl_2_ at 40 °C and pH 6.5 for the times indicated. The enzyme activity against soluble and insoluble rice starch was then assayed in the standard buffer. (**d**) Specific activities of the Y351A variant relative to WT AmyP_ΔSBD_ with soluble and insoluble rice starch as substrates.

We identified two Ca^2+^ ions in the AmyP_ΔSBD_ structure on the basis of high electron densities at their positions and on coordination geometry (Fig. 2a,b). These Ca^2+^ ions, which we designated Ca1 and Ca2, are locate in the periphery of domain A (Fig. 1a). Ca1 lies at the back of the active center, whereas Ca2 lies in the vicinity of the -3 subsite binding loop. Their positions differ from that of the conserved Ca^2+^ at the junction of domains A and B that is present in most GH13 α-amylases.

Ca1 is coordinated by the main-chain oxygens of Asp27, Ile30 and Lys42, and the side-chain oxygens of Asn25 and Asp44, with an average coordinating distance of 2.4 Å (Fig. 2b). This calcium-binding site resembles those in the structures of *B. stearothermophilus* neopullulanase (a GH13_20 family member, pdb accession code 1J0H), *Halothermothrix orenii* α-amylase AmyA (a GH13_36 family member, pdb accession code 1wza^31^) and truncated *Anoxybacillus* α-amylase TASKA (a member of a new GH13 subfamily, pdb accession code 5a2b^20^) (Fig. S3). In particular, the two side-chain carbonyl groups of Asp44 and Asn25 of AmyP_ΔSBD_, both of which function in calcium coordination, are strictly conserved among the four proteins (Figs S2 and S3). Ca2 is coordinated by the main-chain oxygens of Leu348 and Tyr351 and the side-chain oxygens of Asp303, Gln306 and Glu347, with an average coordinating distance of 2.3 Å (Fig. 2a). This calcium-binding site is unique among GH13 α-amylases. Notably, all residues in AmyP_ΔSBD_ that interact with Ca^2+^ through their side chains are conserved among GH13_37 subfamily members (Fig. S2), thus indicating that calcium binding is highly conserved within this subfamily.

To probe the effect of Ca^2+^ ions on enzyme stability, thermal inactivation of the hydrolytic activity of AmyP_ΔSBD_ against both soluble and insoluble rice starch was examined in the presence and absence of added Ca^2+^. AmyP_ΔSBD_ retained more than 80% of its maximal activity after incubation at 40 °C for 10 h in the presence of 10 mM CaCl_2_, in contrast, the enzyme lost most of its activity after incubation at 40 °C for 3 h in the absence of CaCl_2_ (Fig. 2c). These results suggested that Ca^2+^ ions bound to AmyP_ΔSBD_ substantially contribute to the enzyme’s thermostability, as has been found for other GH13 α-amylases. The stabilizing effect of bound Ca^2+^ was ascribed mainly to Ca2, which is adjacent to the -3 subsite. Ca2 helps to maintain the conformations of nearby Arg307 and Tyr351 (Fig. 2a), which interact with their facing residues Asp366 and Val369 through hydrogen bonding and hydrophobic interactions. Asp366 and Val369 are situated in a -3 subsite loop that contains the substrate-binding residues Asp367 and His368. The importance of Tyr351 in stabilizing this -3 subsite loop is supported by the Y351A mutant’s significantly decreased specific activity against both soluble and insoluble rice starch (Fig. 2d) and a greatly increased *K*_m_ for the γ-CD substrate, as compared with the WT enzyme (Table 1). Furthermore, the overall conformation of the Y351A mutant is identical to that of the WT enzyme, as indicated by far-UV CD and tryptophan intrinsic fluorescence spectra (Fig. S4). Together, these results suggest that Ca2 promotes localized stabilization of a -3 subsite loop and that this stabilization helps to preserve the catalytic activity of the enzyme.

### The active site

**Figure 3:**
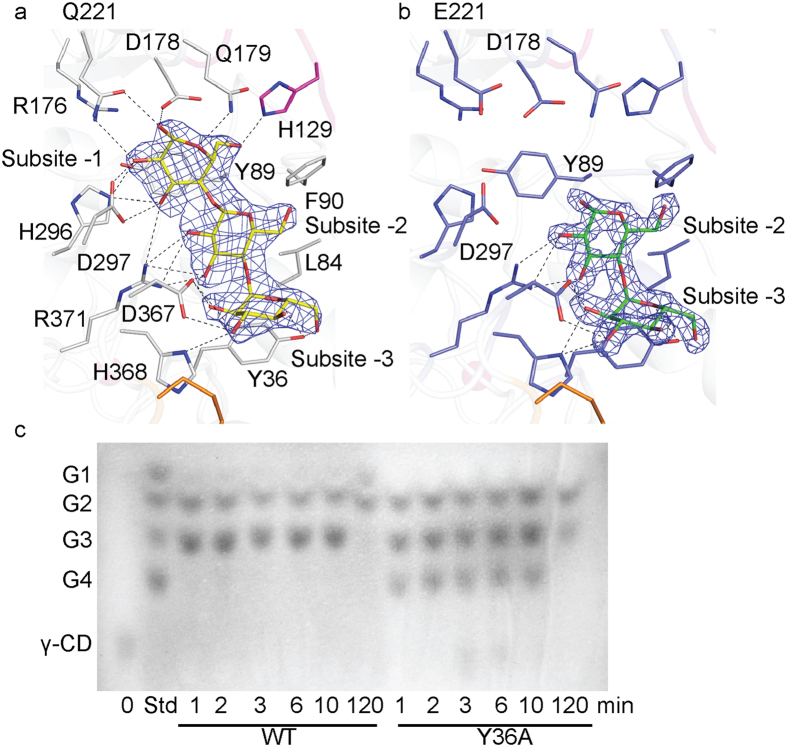
Active site of AmyP_ΔSBD_. (**a**) Electron density of maltotriose bound to the active site of the E221Q mutant of AmyP_ΔSBD_ from the 2*Fo*–*Fc* map contoured at 2σ in the AmyP-E221Q/γ-CD structure. Glucose residues in the oligosaccharide are numbered from the non-reducing end. His129 is from domain B (magenta). Hydrogen bonds are shown as dashed lines. (**b**) Electron density of maltose bound to the active site of WT AmyP_ΔSBD_ from the 2*Fo*–*Fc* map contoured at 2σ in the AmyP/β-CD structure. Glucose residues in the oligosaccharide are numbered from the non-reducing end. (**c**) Time-course analysis of the hydrolysis products of the γ-CD substrate produced by WT AmyP_ΔSBD_ and its Y36A mutant, respectively, using TLC. Std, standards.

The structure of AmyP-E221Q/γ-CD contains electron density representing a maltotriose molecule bound to the active site and occupying subsites -1, -2 and -3 (Fig. 3a), whereas the structure of AmyP/β-CD contains electron density representing a maltose molecule at subsites -2 and -3 (Fig. 3b). These observations suggested that the E221Q mutant possesses activity for γ-CD and the WT enzyme possesses activity for β-CD at the high concentration in the crystallization drop, thus producing maltotriose from γ-CD and maltose from β-CD, respectively.

Structural comparison of the active sites of AmyP_ΔSBD_ and some other homologs in the DALI database indicated the presence of a well-conserved catalytic triad, Asp178, Glu221 and Asp297, located at the center of the barrel (Fig. 1a). Stick superposition of the active site of AmyP_ΔSBD_-E221Q complexed with maltotriose on the structures of GTA (Fig. S5a) and TASKA (Fig. S5b) indicated the remainder of the strictly conserved residues for subsites −1 and −2, including residues participating in hydrogen bonding and aromatic stacking interactions. Owing to its shortened B domain, AmyP_ΔSBD_ lacks residues corresponding to Trp170, Tyr182, Arg220 and His221 for subsites +1 and +2 in GTA. Trp223 of AmyP_ΔSBD_ adopts a conformation identical to that of the corresponding residue in GTA, but differing from that of the corresponding residue in TASKA, which has been proposed to undergo a conformational change after substrate binding^20^. Additionally, in AmyP_ΔSBD_, Gln179 provides a hydrogen bond for subsite -1, and Leu84 provides hydrophobic interactions for subsite -2 (Figs 3a and S5).

Interestingly, the -3 subsite of AmyP_ΔSBD_, which is formed by the hydrogen-bond-forming residues Tyr36, Asp367 and His368, is novel. Tyr36 also stacks against the sugar residue at subsite -3 (Figs 3a,b and S5). This tyrosine and other substrate binding residues of AmyP_ΔSBD_ are all conserved in the GH13_37 subfamily (Fig. S2). Thin-layer chromatography (TLC) of the reaction products showed that the Y36A mutant, compared with the WT, produced a lower ratio of maltotriose (G3) to maltose (G2) during the initial 10 min of hydrolysis with γ-CD as substrate (Fig. 3c). Additionally, the Y36A mutant produced some maltotetrose (G4), whereas the WT enzyme appeared to produce none of this product during the initial stage of hydrolysis (Fig. 3c). This finding may have occured because the Y36A mutant, owing to sharply decreased interaction at subsite -3, was able to cleave G2/maltohexaose (G6) and G3/maltopentaose (G5) from γ-CD, and then G2/G4 and G2/G3 from G6 and G5, respectively. A new but weak substrate-binding site at subsite -4 may be created by the reorientation of nearby residues, such as Trp80, in the Y36A mutant (Fig. S5). This -4 subsite may temporarily protect G4 from rapid digestion. In contrast, the WT enzyme mainly produced G3/G5 from γ-CD, and then G3/G2 from G5, owing to the strong binding force at the -3 subsite. Thus, G3 is the major product of the WT enzyme during the early stage of hydrolysis. This finding confirms the critical role of Tyr36 in subsite -3 binding.

The catalytic and substrate-binding residues in the two structures of AmyP_ΔSBD_ superimposed well. The maltose bound to the active pocket of the WT enzyme also superimposed well at subsites -2 and -3 with the maltotriose bound to the active site of the E221Q mutant (Fig. S6). The observed binding of maltose in subsites -2 and -3 indicated that the affinity of subsite -3 for a glucose residue is greater than that of subsite -1. It has previously been shown that AmyP degrades raw starch, releasing G1, G2 and G3, with complete loss of G4 over time^9^. It seems likely that a strong -3 subsite would protect G3 from rapid digestion, whereas a weak -1 subsite would limit the formation of glucans smaller than G3. To confirm this speculation, the hydrolysis products obtained from 10 mg/ml raw rice starch after incubation with the WT and Y36A mutant AmyP_ΔSBD_ enzymes for various times were analyzed by high-performance liquid chromatography (HPLC). These results (Fig. S7) indicated more efficient production of G3 by the WT enzyme and more efficient production of G2 by the Y36A mutant during the early stage of hydrolysis of insoluble starch, which were generally consistent with the results obtained using γ-CD as substrate. The AmyP_ΔSBD_-E221Q/G3 and AmyP_ΔSBD_/G2 structures can therefore explain the nature of the limit digest of G1, G2 and G3 by the AmyP enzyme.

### Role of a short domain B

**Figure 4:**
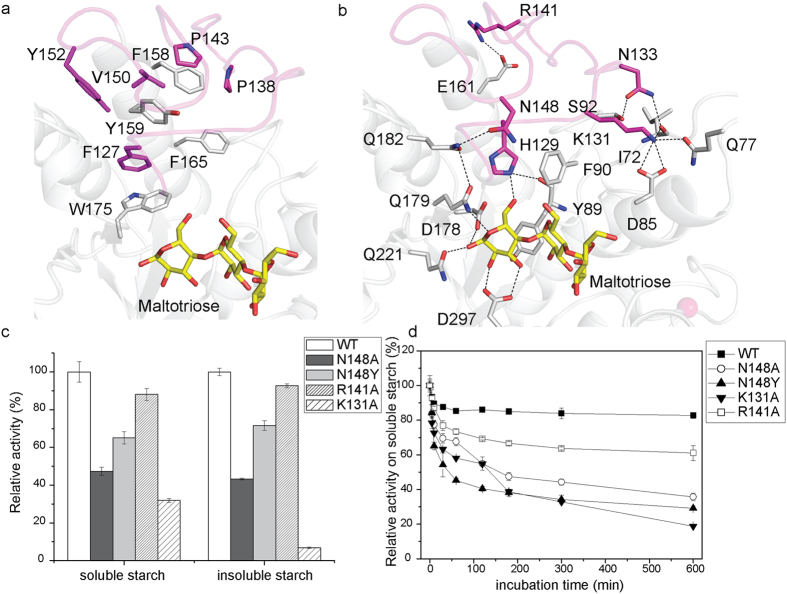
The role of short domain B. (**a**) Stick representation of hydrophobic residues clustered at the interface between domains A and B. (**b**) Stick representation of hydrogen-bond-forming residues between domains A and B. (**c**) Specific activities of the N148A, N148Y, R141A, and K131A variants of AmyP_ΔSBD_ relative to that of WT with soluble and insoluble rice starch as substrates. (**d**) Thermal inactivation of WT AmyP_ΔSBD_ and its N148A, N148Y, K131A, and R141A variants. The enzymes were incubated in the presence of 10 mM CaCl_2_ at 40 °C and pH 6.5 for the times indicated; enzyme activity against soluble rice starch was then assayed in the standard buffer.

In most GH13 α-amylases, the conserved calcium-binding site located between domains A and B is thought to significantly contribute to the protein’s structural stability and to regulate its thermostability and enzymatic activity^7^,^8^,^32^. However, the B domain of AmyP is the smallest among those of GH13 subfamilies of α-amylases, and it does not bind Ca^2+^ or other metal ions. The crystal structures of AmyP_ΔSBD_ show that several hydrophobic residues that are strictly conserved in the GH13_37 subfamily (Fig. S2) cluster at the interface between domains A and B (Fig. 4a). The hydrophobic interactions mediated by these residues are likely to play critical roles in stabilizing the B domain and its interface with domain A. Hydrogen bonds are also involved in the inter-domain interactions within the enzyme (Fig. 4b).

As in other GH13 α-amylases, the small B domain of AmyP_ΔSBD_ is situated adjacent to the active center of the enzyme. To determine the role of the B domain in enzyme catalysis, we generated four site-directed mutants of the B domain, K131A, N148A, N148Y and R141A, and compared their activities and stabilities to those of the WT enzyme (Fig. 4c). The far-UV CD and tryptophan intrinsic fluorescence spectra of all four mutants were essentially identical to those of the WT enzyme (Fig. S8), thus indicating that these mutations did not cause significant changes in the protein’s overall conformation. The K131A mutation had the most profound effect on the enzyme’s activity against soluble and insoluble starch substrates. Kinetic studies revealed that the *K*_m_ value of the K131A mutant for the γ-CD substrate was ten-fold higher than that of the WT enzyme (Table 1), thus indicating a decreased binding affinity for γ-CD. This mutant also showed a considerable decrease in turnover number (*k*_cat_) and catalytic efficiency (*k*_cat_/*K*_m_). The K131A mutant showed markedly decreased activity against soluble starch at pH below 6.5, whereas the WT enzyme and the other mutants retained maximal hydrolytic activity in the pH range of 5.0 to 6.5 (Fig. S9). This phenomenon may have been because the replacement of Lys131 with an alanine significantly decreased the rigidity of the -1 subsite binding loop (residues 128–133) in domain B (Fig. 3a) because of the absence of the four hydrogen bonds nomally formed by Lys131 with domain A (Fig. 4b); this change probably perturbs the proper conformation of the substrate-binding residue His129. It was noted that the N148A mutant displayed a greater decrease in activity against soluble and insoluble starch substrates than the N148Y mutant (Fig. 4c). Moreover, for the degradation of γ-CD, the N148A variant, compared with that of the N148Y mutant, exhibited a larger decrease in *k*_cat_/*K*_m_ (Table 1). These results suggest that Asn148 lines one side of the entrance to the active site. Mutations at this residue disrupt the network of hydrogen bonds formed by Asn148, Gln182, Gln179 and the sugar residue at subsite -1 (Fig. 4b) and thus decrease the enzyme’s catalytic efficiency. However, the substitution of Asn148 by Tyr might introduce extra stacking interactions of this residue with nearby residues such as His129, Phe90 and/or substrate sugar rings, thus disorganizing the architecture of the active-site residues to a lesser extent than occurs in the N148A mutation. The R141A mutant and WT AmyP_ΔSBD_ displayed similar levels of activity against soluble and insoluble rice starch (Fig. 4c), possibly because Arg141, which is located opposite the catalytic region, had only a slight effect on catalysis.

Thermal inactivation studies showed that the activity of WT AmyP_ΔSBD_ against both soluble (Fig. 4d) and insoluble starch substrates was more thermostable than that of the four mutants (Fig. S10), thus demonstrating the importance of the rigidity of domain B in the thermostablity of the enzyme. Together, these results suggest that, despite its small size, the B domain of AmyP_ΔSBD_ has a function similar to those of the B domains of other GH13 α-amylases, that is, to reinforce the conformation of the active site and to enhance the enzyme’s thermostability.

## Discussion

The crystal structures of AmyP_ΔSBD_ presented in this work provide the first insights into the overall structure, Ca^2+^ binding sites, and substrate-binding subsites of α-amylases belonging to the new subfamily GH13_37. A key feature of these structures is the very short length of its B domain, a trait shared by GH13_37 subfamily members (Fig. S2). To our knowledge, the B domain of AmyP_ΔSBD_ is the smallest B domain in presently known GH13 α-amylases. This B domain is formed exclusively by loop elements, does not bind metal ions, and helps to form a wide-open entrance to the catalytic cleft. In the case of AmyP_ΔSBD_, hydrophobic interactions and hydrogen bonds play important roles in the association of domains A and B. Like B domains of other GH13 α-amylases, the B domain of AmyP_ΔSBD_, which is located in proximity to the catalytic region, contributes to the thermostability and activity of the enzyme. Although a common calcium-binding site at the interface between the A and B domains is not present in AmyP_ΔSBD_, two calcium-binding sites are found, one of which is unique and adjacent to the -3 subsite binding loop in domain A. The calcium binding in AmyP_ΔSBD_ significantly enhances the enzyme’s thermostability. In this work, the resulting glucan in the crystal structures, however, was not the cyclodextrin, but a degradation product, G2 in the case of the WT enzyme and G3 in the case of the active-site mutant. The observation that G2 binds in subsites -2 and -3 indicates tighter binding in these subsites than in subsite -1, which is consistent with the production of a limit digest of G1, G2 and G3 by the enzyme. The novel Tyr36 of AmyP participates in both stacking and hydrogen-bonding interactions with the sugar residue at subsite -3. The importance of Tyr36 in the -3 subsite binding was confirmed by the significantly increased ratio of G2 to G3 produced by the Y36A mutant from both γ-CD and insoluble starch substrates during the early stage of hydrolysis. The information presented here on the new GH13 subfamily may aid in engineering the thermostability, calcium requirements and product composition of α-amylases for industrial applications.

**Figure 5:**
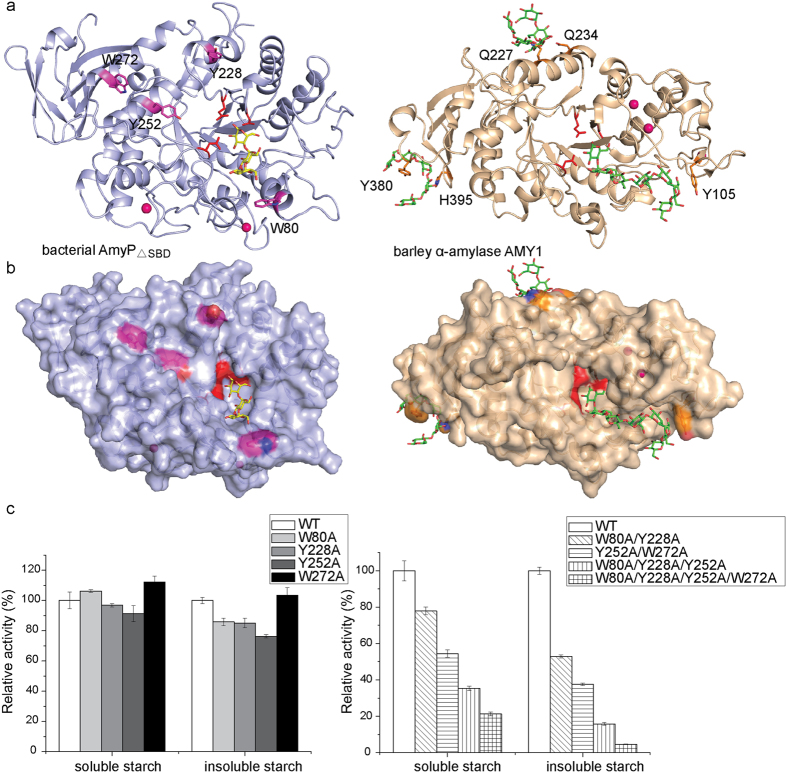
The roles of surface aromatic residues. (**a**) Stick representation of the surface aromatic residues (magenta) of AmyP_ΔSBD_ (blue on the left) and the surface aromatic residues (orange) of barley α-amylase AMY1 (pdb accession code 1RP8, light orange on the right). Saccharide bound in the active site cleft of AmyP_ΔSBD_ is shown as yellow sticks. Saccharides bound in the active site cleft and at surface sites of AMY1 are represented by green sticks. In both structures, the catalytic residues are shown in red and calcium ions are shown as hot pink spheres. (**b**) Surface representation of AmyP_ΔSBD_ (left) and barley α-amylase AMY1 (right). (**c**) Comparison of the specific activities of WT AmyP_ΔSBD_, its W80A, Y228A, Y252A, and W272A single point mutants (left) and its W80A/Y228A, Y252A/W272A, W80A/Y228A/Y252A, and W80A/Y228A/Y252A/W272A multiple point variants (right) using soluble rice starch and insoluble rice starch, respectively, as substrates.

Here, we described the crystal structure of a bacterial RSDE. RSDEs may exhibit similar structural properties that are related to their ability to digest raw starch. The structural comparison of AmyP_ΔSBD_ and GTA reveals that AmyP_ΔSBD_ lacks a structural lid above the active site, owing to the short length of its B domain. GTA may also be a RSDE, because it has an identical protein sequence to that of Pizzo extracellular α-amylase, which can adsorb onto and hydrolyze raw starch^21^,^22^. However, GTA does not possess a C-terminal domain rich in aromatic residues, whereas Pizzo α-amylase has been studied in the presence of this extended region. It has been reported that the B domain of GTA is one of the smallest B domains found in α-amylases^21^. In contrast, the domain B of AmyA has an elongated architecture, which creates a structural lid above the active site over a wider area and probably restricts substrate access to a larger extent (Figs S2 and S11). Structural comparison of AmyP_ΔSBD_ and the well-studied RSDE barley α-amylase AMY1 (pdb accession code 1RP8^16^) shows that both of their B domains point away from the catalytic site, forming a much wider cleft allowing the substrate to enter (Fig. 5). Therefore, enhanced accessibility of the catalytic cleft may be necessary for the enzyme to degrade raw starch granules.

Aromatic residues are central in protein-carbohydrate interactions. Because AmyP_ΔSBD_ has several aromatic residues exposed on its surface at a certain distance from the active site, we hypothesized that these residues might enable stacking interactions with glucose motifs and enhance the enzyme’s activity against starch. To test this hypothesis, four surface aromatic residues (Fig. 5) were mutated to alanine to generate eight mutants: four single mutants (W80A, Y228A, Y252A and W272A), two double mutants (W80A/W228A, Y252A/W272A), one triple mutant (W80A/Y228A/Y252A) and one quadruple mutant (W80A/Y228A/Y252A/W272A). The aromatic side chains of Tyr228/Trp272 and Trp80/Tyr252 are perpendicular and parallel to the protein surface, respectively (Fig. 5). In addition, Trp80, Tyr252 and Trp272 are conserved in the GH13_37 subfamily (Fig. S2). The far-UV CD and tryptophan intrinsic fluorescence spectra of the mutant proteins indicated that the overall conformation of AmyP_ΔSBD_ was still retained in all mutants (Fig. S12). It appeared that a single mutation did not reduce the enzyme’s specific activity against soluble or insoluble rice starch to any significant extent. Nevertheless, multiple point mutants at surface aromatic residues resulted in pronounced decreases in starch hydrolytic activity. The quadruple mutant was the most significantly altered; its activities against soluble and insoluble rice starch were approximately 20% and 5%, respectively, of those of the WT enzyme. These results indicated that these exposed aromatic residues may together facilitate the attachment of the enzyme to glucan substrates; the resulting absence of saccharide-binding ability at these secondary binding sites in the quadruple mutant resulted in a significant reduction in the enzyme activity. A similar synergistic effect has been observed in other RSDEs such as AMY1^15^,^33^ and human salivary α-amylase^19^. However, this feature may not be as obvious in AmyP_ΔSBD_ as it is in AMY1, because saccharide binding at SBSs is directly seen in the crystal structures of AMY1^16^,^17^, but not in those of AmyP_ΔSBD_. This feature of AmyP_ΔSBD_ is consistent with its weak binding to raw starch granules. In general, RSDEs often possess extra starch-binding regions formed by surface aromatic residues that enhance their enzymatic activity.

The attachment of AmyP to starch granules is mainly ascribed to its C-terminal SBD domain. This SBD belongs to a new carbohydrate-binding-module family, CBM69. Further biochemical work is required to determine the structure-function relationship of this novel SBD. Two cysteine residues flank the N and C termini of the SBD and both of these residues are highly conserved in the GH13_37 subfamily. These two cysteines may be involved in a disulfide bridge that enhances the conformational stability of the SBD. Improvement of the *E. coli* expression of the SBD domain of AmyP to facilitate correct protein folding is currently being investigated by our group.

## Methods

### Cloning, expression and purification

The pET32a-amyp plasmid encoding full-length AmyP (GenBank accession number HM572234) was used as a PCR template. DNA fragments corresponding to full-length AmyP (residues 1–639), the SBD-truncated mutant AmyP_ΔSBD_ (residues 1–496) and the SBD (residues 491–639) were amplified by PCR and cloned into a modified pET-28a(+) vector in which the thrombin protease site was substituted for the tobacco etch virus (TEV) cleavage site. All of the mutants were generated with a Mutant Best kit (Takara). All clones were verified by DNA sequencing.

Recombinant AmyP and AmyP_ΔSBD_ were transformed into *E. coli* BL21 (DE3) cells. The transformed cells were grown at 37 °C to an OD_600_ of approximately 0.8 before induction with 0.2 mM isopropyl-β-D-thiogalactopyranoside. The culture was incubated at 16 °C for an additional 24 h before the cells were harvested and lysed by sonication.

Full-length AmyP and its E221Q mutant derivative were purified for crystallization using a HisTrap column (GE Healthcare), treated with TEV to remove the 6 × His tag at the N-terminus and was further purified on a HisTrap column and then subjected to size-exclusion chromatography on a HiLoad 16/60 Superdex 200 column (GE Healthcare) and anion-exchange chromatography on a MonoQ HR 5/5 column (GE Healthcare). The purified proteins were dialyzed against buffer A (20 mM Tris-HCl [pH 8.0], 0.5 M NaCl) and concentrated. AmyP_ΔSBD_ and all mutant proteins used in enzyme activity assays, as well as the SBD of AmyP used in CD spectrum examination, were purified using a HisTrap column, and subsequent on a HiLoad 16/60 Superdex 200 column equilibrated with buffer A.

### Crystallization, data collection and structure determination

All crystals were grown using the sitting drop vapor diffusion method at 10 °C. Crystal screen I and II kits (Hampton Research) were used to identify initial crystallization conditions. The conditions were further optimized to obtain good diffraction-quality crystals. Crystals of the E221Q mutant of full-length AmyP co-crystallized with γ-CD (AmyP-E221Q/γ-CD) were obtained by mixing equal volumes of a 5 mg/ml protein sample pre-incubated with 10 mM γ-CD and a reservoir solution containing 0.1 M sodium citrate [pH 6.0], 22.5% (v/v) 2-propanol, and 22.5% (w/v) polyethylene glycol (PEG) 4000. Crystals of WT full-length AmyP co-crystallized with β-CD (AmyP/β-CD) were grown using a 7 mg/ml protein solution pre-incubated with 4 mM β-CD and then diluted 1:1 with reservoir solution containing 0.1 M Tris-HCl [pH 8.5], 0.2 M sodium acetate, and 30% (w/v) PEG 4000.

All crystals used for diffraction data collection were quickly transferred to a cryoprotectant solution consisting of 25% glycerol (v/v) in reservoir solution, and were flash-frozen in liquid nitrogen. The data sets of AmyP-E221Q/γ-CD and AmyP/β-CD were collected on beam line 17U (BL17U) at the Shanghai Synchrotron Radiation Facility (SSRF).

The data sets were indexed, integrated and scaled with HKL2000^34^. The AmyP-E221Q/γ-CD structure was solved by molecular replacement with Phaser^35^ in the CCP4^36^ software suite by using the *B. stearothermophilus* neopullulanase (pdb accession code 1J0H) as a search model, autobuilt with ArpWarp^37^ and refined and manually built using Refmac5^38^ and COOT^39^. The AmyP/β-CD structure was then solved using AmyP-E221Q/γ-CD as a model. The crystal diffraction data and refinement statistics are listed in Table 2.

### Enzyme activity assays

The starch-hydrolyzing activities of the WT and E221Q mutant AmyP enzymes were measured at 40 °C for 5 min in buffer B (20 mM HEPES [pH 6.5], 150 mM NaCl, 10 mM CaCl_2_) using 10 mg/ml soluble starch as substrate, which was freshly prepared by dissolving soluble starch powder in buffer B with brief heating and then cooling to room temperature. The enzyme reactions were initiated by the addition of 0.05 ml of 5 μg/ml WT or 1 mg/ml E221Q mutant AmyP enzyme to 0.55 ml of pre-heated soluble starch solution. The starch-hydrolyzing activities of the WT and mutant AmyP_ΔSBD_ enzymes were measured at 40 °C for 5 min in buffer B using 10 mg/ml soluble starch and 10 mg/ml insoluble rice starch as substrates. Raw rice starch granules were washed three times with ultrapure water followed by buffer B, suspended in buffer B with shaking, and used immediately. Reactions were initiated by the addition of 0.05 ml of 2 μg/ml WT or 2–10 μg/ml mutant AmyP_ΔSBD_ enzyme to 0.55 ml pre-heated soluble starch solution, or by the addition of 0.05 ml of 20 μg/ml WT or 20–100 μg/ml mutant AmyP_ΔSBD_ enzyme to 0.55 ml raw rice starch solution. The reducing sugars produced in the reaction were quantified using the 3,5-dinitrosalicylic acid (DNS) method^40^. The absorbance at 540 nm was measured and the amount of reducing sugar present was determined on the basis of a standard curve using glucose. The enzyme concentration was determined using the Bradford method. All experiments were performed in triplicate. The average and standard deviation values were calculated; the specific activity of the WT enzyme was taken as 100% for determining relative activities.

### Enzyme kinetics

Reactions for assessing kinetics were conducted at 40 °C in buffer B using γ-CD as substrate at nine different concentrations. Reactions were initiated by the addition of 0.05 ml of 1 μg/ml WT or 1–5 μg/ml mutant AmyP_ΔSBD_ enzyme to 0.45 ml γ-CD solution. Reducing sugar quantities were determined using the copper-bicinchoninate (CuBic) method^41^. The absorbance at 570 nm was measured; and the corresponding amount of reducing sugar was determined via a standard curve using glucose. The initial velocity of the reaction was determined by plotting absorbance versus time for each substrate concentration. The kinetic parameters *k*_cat_, *K*_m_, and *k*_cat_/*K*_m_ were calculated by fitting initial velocities versus substrate concentrations to the Michaelis-Menten equation using nonlinear regression with Origin.

### Enzyme thermal inactivation

The effect of Ca^2+^ ions on the thermal inactivation of the enzyme was evaluated by incubating WT AmyP_ΔSBD_ at 40 °C in 20 mM HEPES [pH 6.5] and 150 mM NaCl with or without 10 mM CaCl_2_. Comparison of the thermal inactivation of WT and mutant AmyP_ΔSBD_ was conducted by incubating the enzymes at 40 °C in buffer B; the residual activity was then determined at intervals with the DNS method using 10 mg/ml soluble starch and 10 mg/ml insoluble starch as substrates.

### TLC of hydrolysis products

Hydrolysis products were detected by TLC after incubation of the WT and Y36A mutant AmyP_ΔSBD_ enzymes with the γ-CD substrate for various times. Activity assays were performed at 40 °C in buffer C (20 mM HEPES [pH 6.5], 50 mM NaCl, 10 mM CaCl_2_). Each reaction contained 0.1 ml of 3 mg/ml enzyme and 0.9 ml of 10 mg/ml γ-CD solution. Three microliters of each reaction was blotted onto a 20 cm × 20 cm, 500-mm-thick silica gel 60 A˚ plate (Qingdao Haiyang Chemical Co. Ltd., China). The plate was dried and placed in a chamber containing a mixture of isopropanol:ethyl acetate:water (3:1:1, v/v/v). The plate was dried, and the contents were visualized by dipping the plate into a solution containing 0.3% N-(1-naphthyl)-ethylenediamine and 5% H_2_SO_4_ in methanol and heating for 10 min at 110 °C as described by Shim et al^42^. Standards (1 μl each of a 10 mg/ml solution) were also blotted on the plate for comparison and determination of the reaction products.

### HPLC of hydrolysis products

The hydrolysis products against raw rice starch after incubation with the WT and Y36A mutant AmyP_ΔSBD_ enzymes for various times were identified with HPLC. Activity assays were performed at 40 °C in buffer C. Each reaction was initiated by the addition of 0.1 ml of 1 mg/ml enzyme to 0.9 ml of 10 mg/ml raw rice starch solution and was terminated by heating at 100 °C for 10 min. The mixtures were then centrifuged at 10,000 × g for 10 min. Each supernatant was diluted 10-fold with ultrapure water, and 10 μl of the diluted supernatant was applied to an HPLC system (Agilent 1260 Series). The column (TSK-GEL amide-80 column (4.6 × 250 mm; Tosoh, Tokyo, Japan) was equilibrated with 55% acetonitrile. The components were separated in 55% acetonitrile at a flow rate of 1 ml/min at room temperature. The yields of sugar molecules were analyzed using an evaporative light scattering detector incorporated into the HPLC system. Glucose, maltose, maltotriose and maltotetraose (20 μl each of a 5 mM solution) were used as standards.

### Far-UV CD spectra

Far-UV CD spectra were recorded at 25 °C with a MOS-500 spectropolarimeter, at wavelengths between 200 and 250 nm, using a 0.1-cm path length cell and approximately 0.2 mg/ml protein sample in buffer D (0.15 M NaH_2_PO_4_/Na_2_HPO_4_ [pH 7.5], 50 mM NaCl). A buffer only reference was subtracted from each curve.

### Intrinsic fluorescence spectra

Intrinsic fluorescence measurements of proteins were recorded at 25 °C with a F-4500 spectrofluorophotometer in a 0.1-cm path length quartz cell using approximately 0.2 mg/ml protein sample in buffer A. Fluorescence emission spectra were recorded from 300 nm to 400 nm using a maximum excitation wavelength of 288 nm.

## Additional Information

**Accession codes:** The atomic coordinates and structure factors for the E221Q mutant of full-length AmyP co-crystallized with γ-CD (AmyP-E221Q/γ-CD) and full-length AmyP co-crystallized with β-CD (AmyP/β-CD) have been deposited in the Protein Data Bank under accession codes 5H05 and 5H06, respectively.

**How to cite this article:** Liu, Y. *et al*. Crystal structure of a raw-starch-degrading bacterial α-amylase belonging to subfamily 37 of the glycoside hydrolase family GH13. *Sci. Rep.* **7**, 44067; doi: 10.1038/srep44067 (2017).

**Publisher's note:** Springer Nature remains neutral with regard to jurisdictional claims in published maps and institutional affiliations.

## Supplementary Material

Supplementary Information

## Figures and Tables

**Table 1:** Kinetic parameters of the WT and mutant AmyP_ΔSBD_ enzymes for the γ-CD substrate.

Enzyme	*K*_m_ (mM)	*k*_cat_ (s^−1^)	*k*_*cat*_/*K*_m_ (s^−1^ mM^−1^)
WT	0.12 ± 0.005	39.58 ± 0.004	323.2
N148Y	0.10 ± 0.009	26.11 ± 0.01	273.7
N148A	0.20 ± 0.03	22.59 ± 0.007	112.8
Y351A	2.11 ± 0.09	25.02 ± 0.006	11.9
K131A	1.29 ± 0.13	7.47 ± 0.004	5.8

**Table 2:** Data collection and refinement statistics for AmyP/β-CD and AmyP-E221Q/γ-CD.

Structure	AmyP/β-CD	AmyP-E221Q/γ-CD
Wavelength (Å)	0.9793	0.9793
Space group	P2_1_2_1_2_1_	P2_1_
Cell parameters		
a, b, c (Å)	79.87, 130.53, 217.53	79.87, 130.53, 217.53
α, β, γ (°)	90.00, 90.00, 90.00	90.00, 93.14, 90.00
Resolution (Å)	40.00–1.95 (2.02–1.95)^a^	40.00–2.55 (2.64–2.55)
*R*_merge_ (%)	10.9(47.4)	12.1(64.2)
I/σI	25.5(4.0)	11.78(2.0)
Completeness (%)	87.6(85.0)	99.7(99.9)
Redundancy	5.0(5.2)	3.5(3.5)
Refinement		
No.reflections (overall)	144717	95368
No. reflections (test)	7292	4716
*R*_work_/*R*_free_ (%)	16.35/21.23	17.02/22.00
No. non-H atoms		
Protein	15550	15498
Sugar	92	136
Ca1/Ca2	4/4	4/4
Water	2177	431
B factors (Å^2^)		
Protein	13.96	37.20
Sugar	14.57	33.49
Ca1/Ca2	28.31/10.89	59.98/57.93
Water	22.05	36.34
R.m.s. deviations		
Bond lengths (Å)	0.006	0.008
Bond angles (°)	0.828	0.933
Rampage plot% residues		
Favored	97.28	96.32
Allowed	2.52	3.33
Outliers	0.20	0.35

^a^Values in parentheses are for the highest-resolution shell.
