# Supplementary material for: Crystal structure of a raw-starch-degrading bacterial α-amylase belonging to subfamily 37 of the glycoside hydrolase family GH13
Source: Sci Rep. 2017 Mar 17;7:44067. doi: 10.1038/srep44067 (PMC5355875; doi:10.1038/srep44067)

Crystal structure of a raw starch-degrading bacterial α-amylase belonging to subfamily 37 of glycoside hydrolase family GH13

Yanhong Liu[a], Jigang Yu[a], Fudong Li[b], Hui Peng[a], Xuecheng Zhang[a], Yazhong Xiao[a], Chao He[a*]

[a]Anhui Provincial Engineering Technology Research Center of Microorganisms and Biocatalysis and School of Life Sciences, Anhui University, Hefei, Anhui 230601, China

[b]Hefei National Laboratory for Physical Sciences at Microscale and School of Life Sciences, University of Science and Technology of China, Hefei, Anhui 230026, China

[*]Correspondence to Chao He, Tel.: (86) 0551-63861928, E-mail: chaohe@ahu.edu.cn

Supplementary Information

Fig. S1

(a) Several crystals of AmyP-E221Q/γ-CD were washed stepwise in three droplets of reservoir solution and then dissolved in a droplet of protein buffer A. The crystal washing and dissolving droplets were analyzed with SDS-PAGE: (lane 2-4) the remaining droplets after washing the crystals; (lane 5) the dissolved crystals; (lane 6-7) the purified full-length AmyP and AmyP$_{\Delta SBD}$ protein solutions; (lane 1, 8) protein molecular weight marker. Peptide sequencing of protein bands from the dissolved crystal sample was performed by LC-MS. (b) Far-UV CD spectrum for the SBD domain of AmyP.

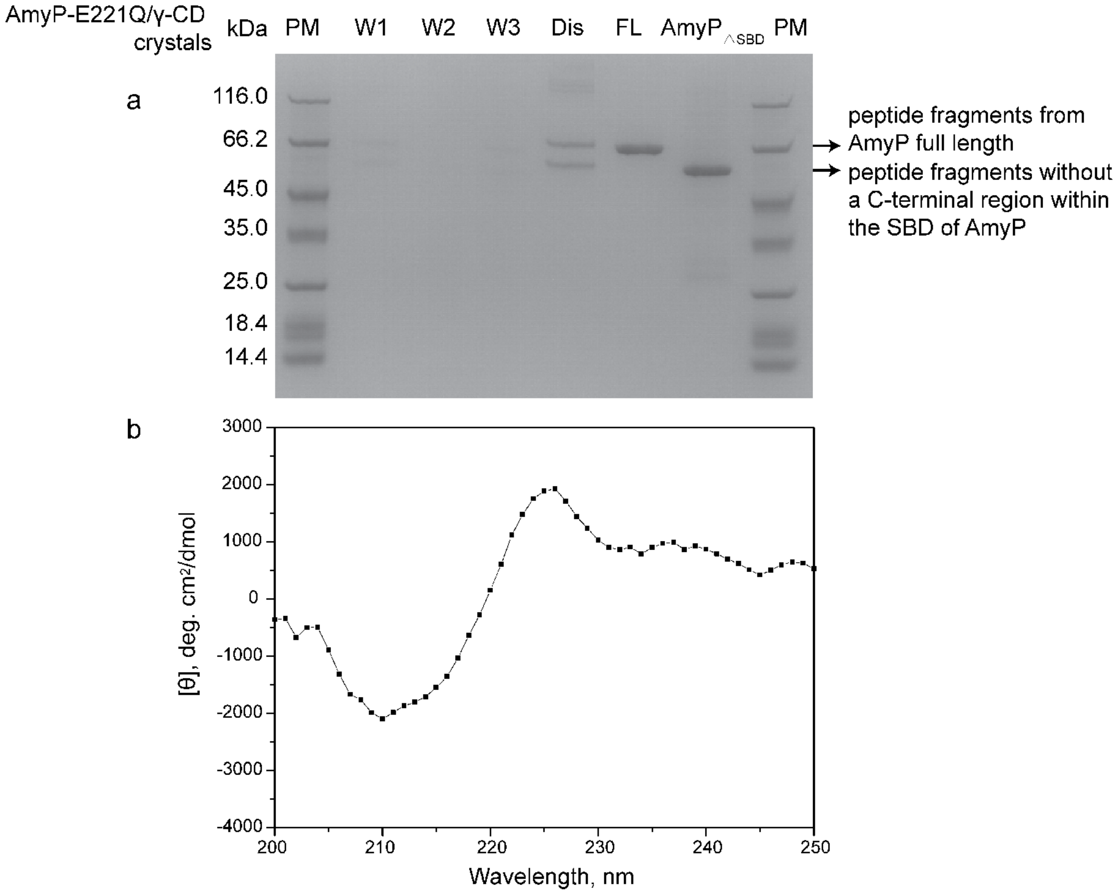

Fig. S2

Multiple sequence alignment of AmyP$_{\Delta SBD}$ with other GH13_37 subfamily members, and the *B. stearothermophilus* neopullulanase (pdb accession code 1J0H), *H. orenii* α-amylase AmyA (pdb accession code 1wza), *G. thermoleovorans* α-amylase GTA (pdb accession code 4e2o), truncated *Anoxybacillus* α-amylase TASKA (pdb accession code 5a2b), and barley α-amylase AMY1 (pdb accession code 1RP8). Sequences corresponding to domain B are indicated with a box in magenta. Cysteine residues conserved in the GH13_37 subfamily are marked by numbers below. Ca^{2+}-interacting residues through their side chains of AmyP$_{\Delta SBD}$ are labeled by green circles. Catalytic and substrate binding residues of AmyP$_{\Delta SBD}$ are labeled by red and yellow squares, respectively. Several hydrophobic and hydrogen-bond interacting residues at the interface between domains A and B are labeled by purple and orange triangles, respectively. Surface aromatic residues are highlighted by blue stars.

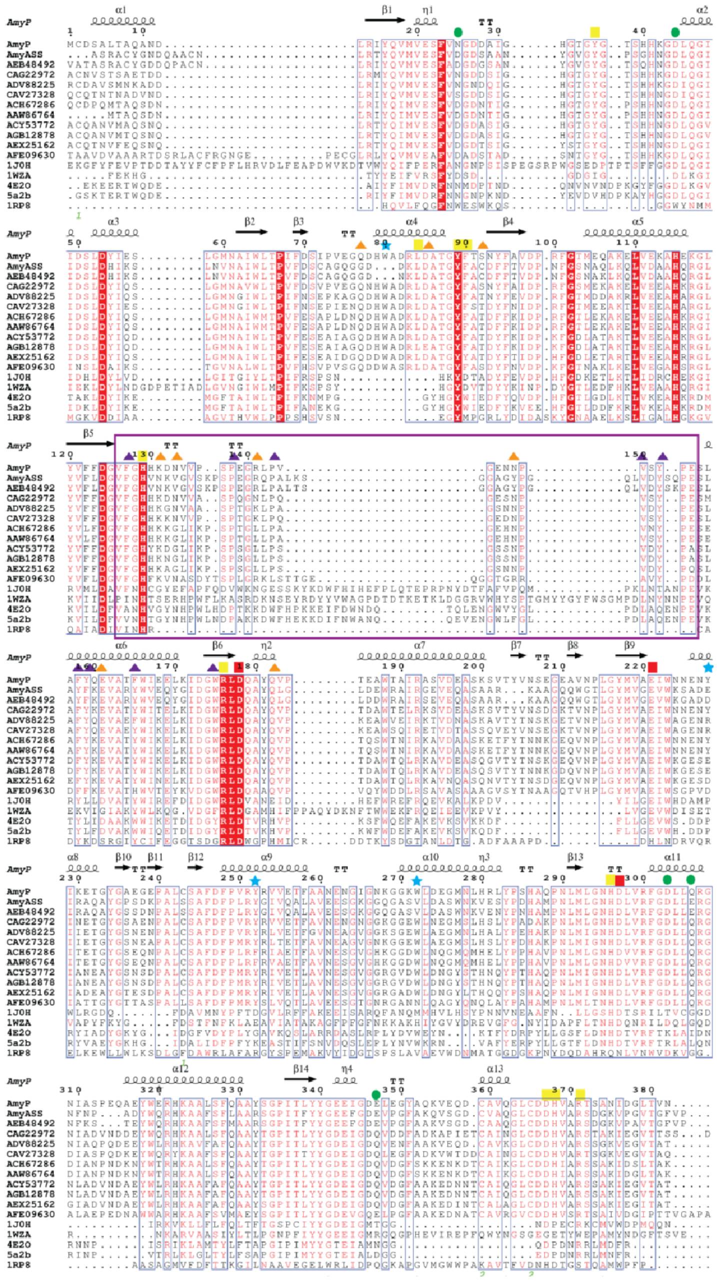

Fig. S3

(a) Similar calcium binding sites were observed in the superimposed structures of AmyP$_{\Delta SBD}$ (gray), the *B. stearothermophilus* neopullulanase (pink, pdb accession code 1J0H), *H. orenii* α-amylase AmyA (cyan, pdb accession code 1wza), and truncated *Anoxybacillus* α-amylase TASKA (pdb accession code 5a2b). The conserved Ca^{2+} binding residues are labeled. The residue numbers in parentheses refer to the *B. stearothermophilus* neopullulanase, AmyA and TASKA, respectively. The calcium binding sites in the *B. stearothermophilus* neopullulanase, AmyA and TASKA are shown in (b), (c) and (d), respectively.

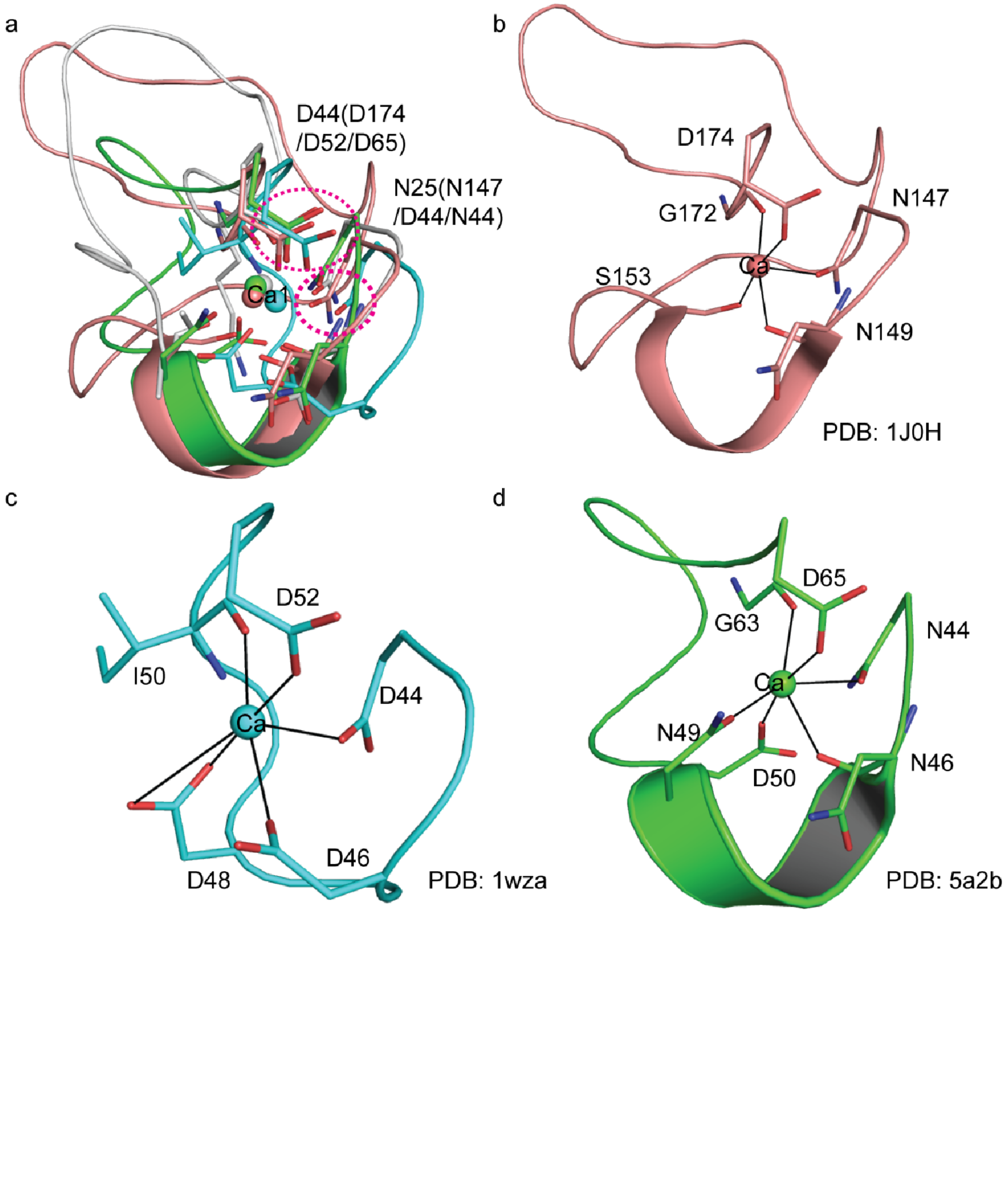

Fig. S4

(a) Far-UV CD and (b) intrinsic fluorescence emission spectra for WT AmyP$_{\Delta SBD}$ and its Y351A variant were recorded.

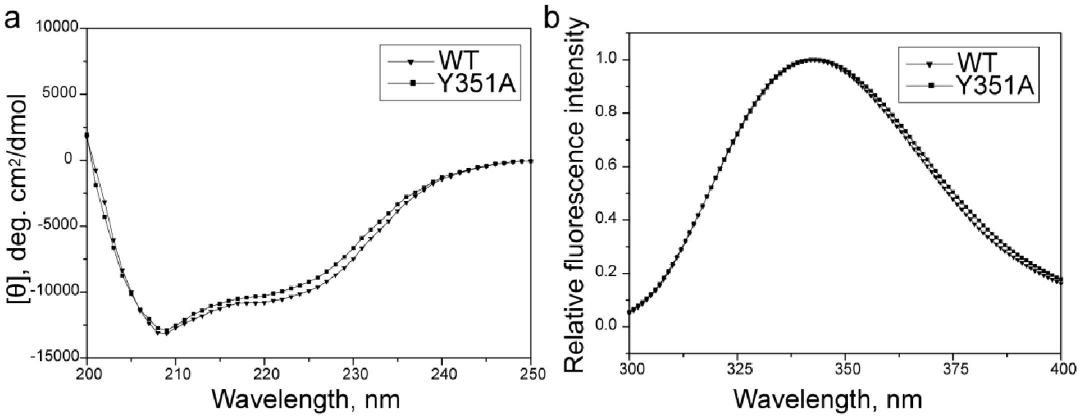

Fig. S5

Stick superposition of the active sites of the E221Q mutant of AmyP$_{\Delta SBD}$ (gray) in complex with maltotriose (yellow) on GTA (orange, pdb accession code 4e2o) (a) and TASKA (green, pdb accession code 5a2b) (b), respectively. Glucose residues in the oligosaccharide are numbered from the non-reducing end. Conserved and nonconserved substrate binding residues of AmyP$_{\Delta SBD}$ are labeled in black and magenta, respectively. The residue numbers in parentheses refer to GTA and TASKA.

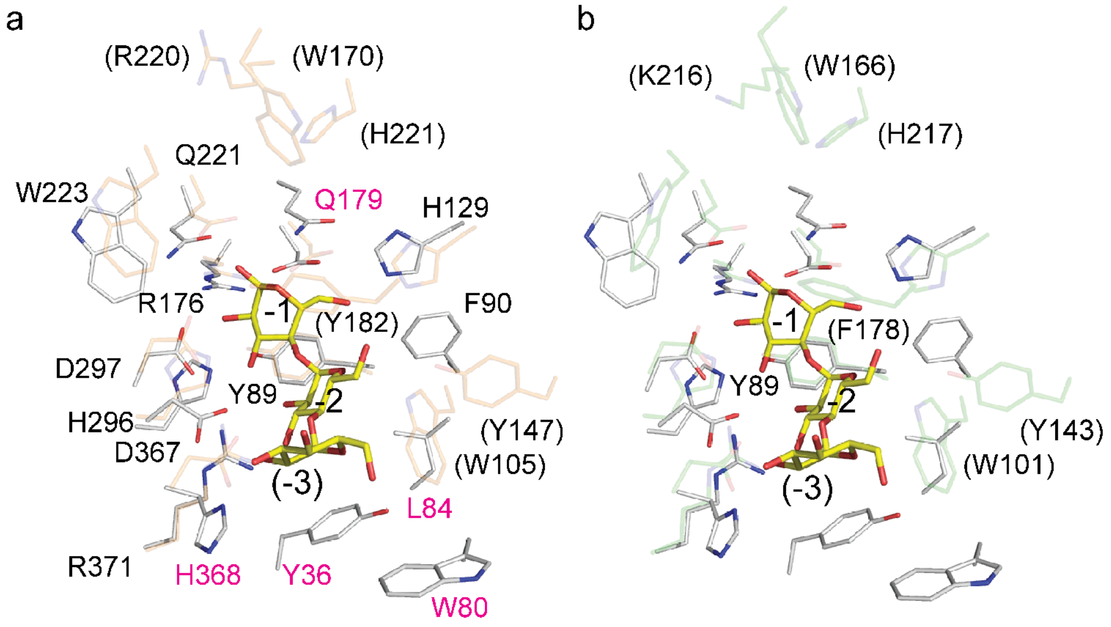

Fig. S6

Stick superposition of the substrate binding residues of the E221Q mutant complexed with maltotriose and the WT complexed with maltose. Glucose residues in the oligosaccharide are numbered from the non-reducing end.

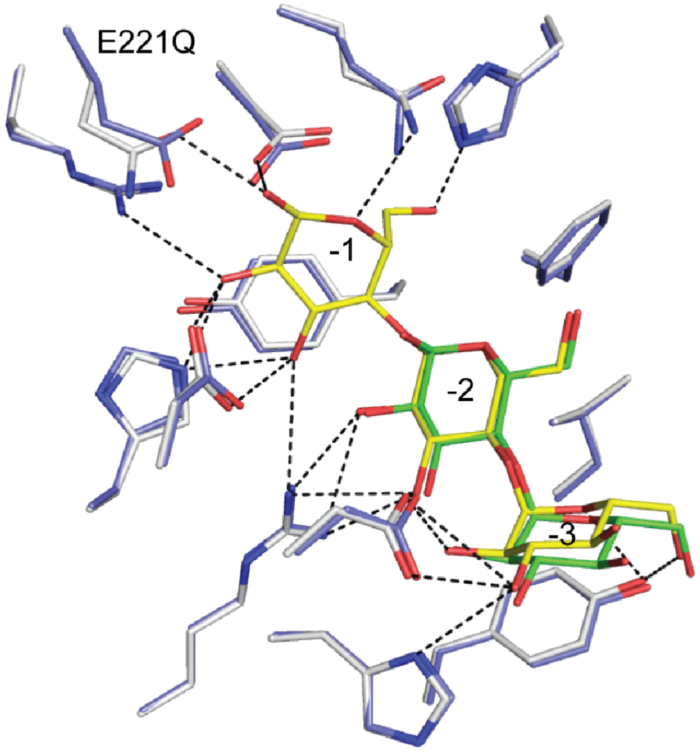

Fig. S7

Time-course analysis of the hydrolysis products for 10 mg/ml raw rice starch produced by WT AmyP$_{\triangle SBD}$ and its Y36A mutant using HPLC.

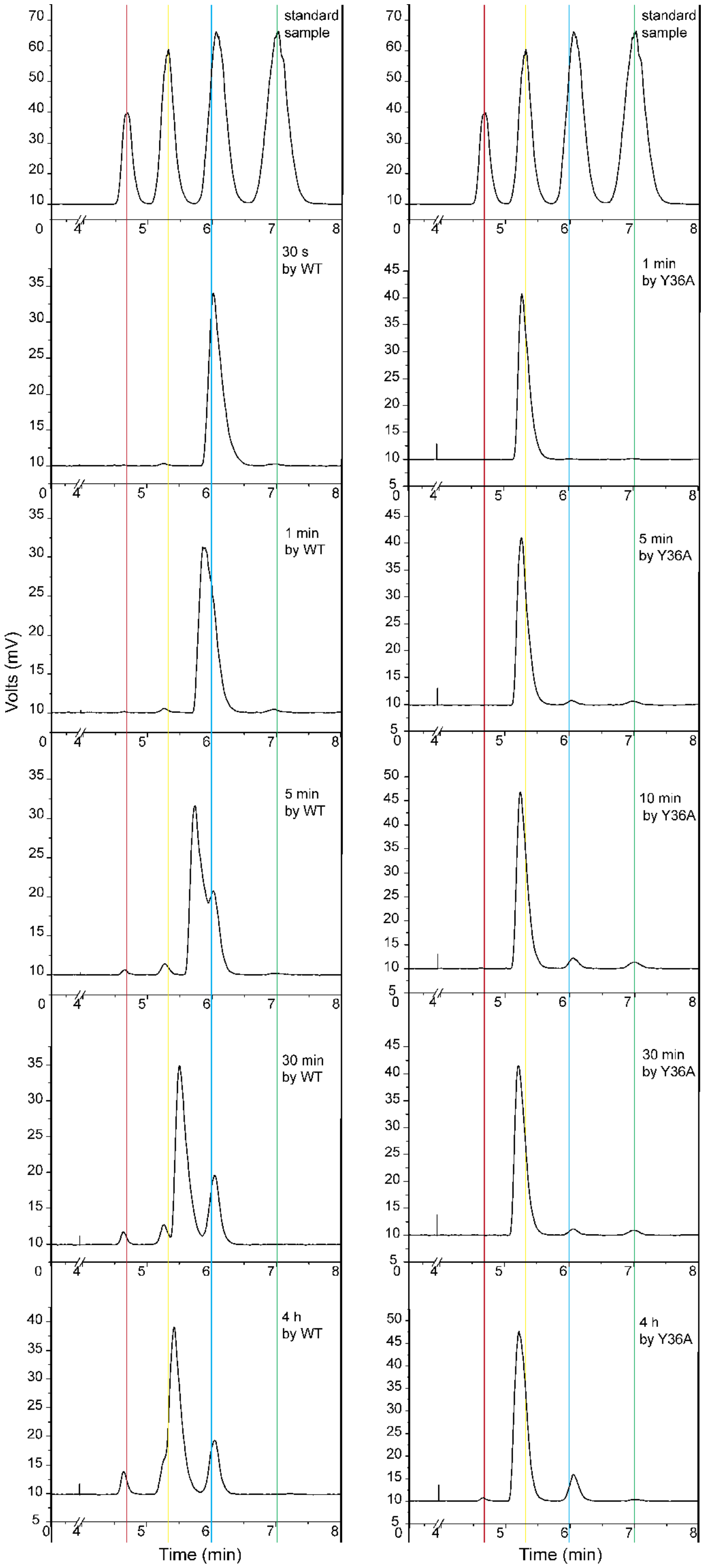

Fig. S8

(a) Far-UV CD and (b) intrinsic fluorescence emission spectra for WT AmyP$_{\Delta SBD}$ and its K131A, N148Y, N148A and R141A variants on domain B were recorded.

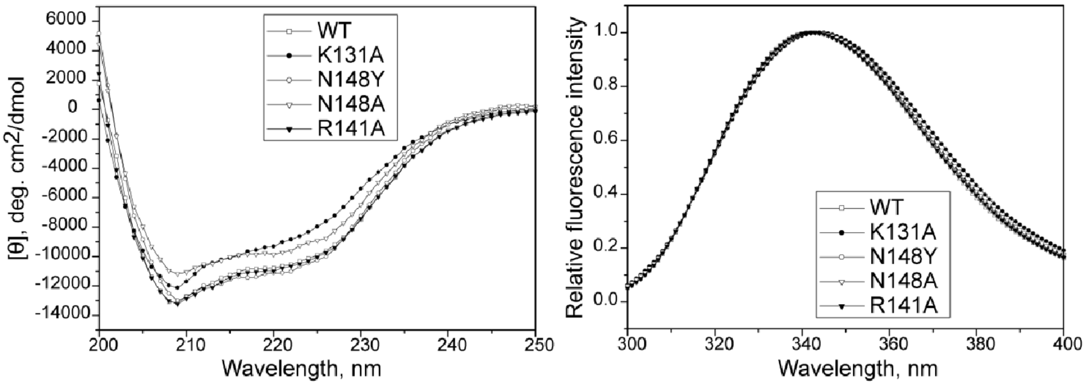

Fig. S9

Effects of pH on activites of WT AmyP$_{\Delta SBD}$ and its K131A, N148A, N148Y and R141A variants on soluble rice starch.

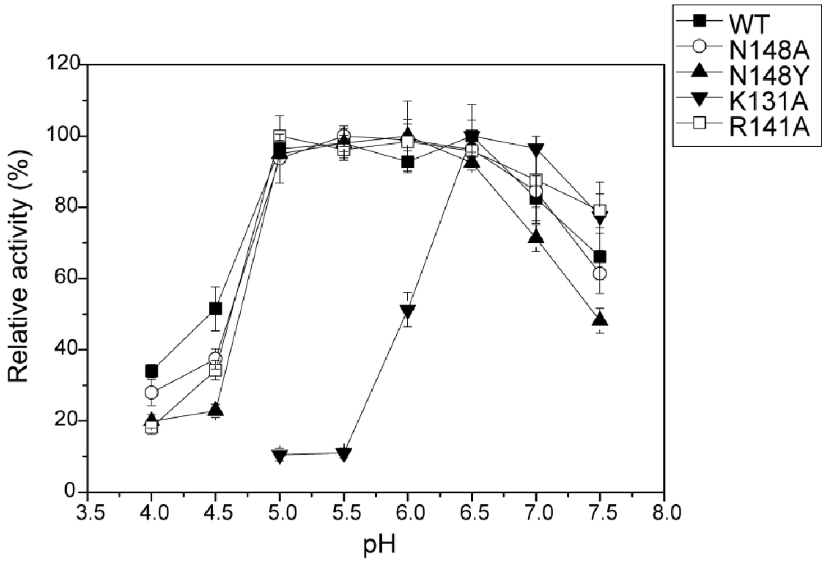

Fig. S10

Thermal inactivation of WT AmyP$_{\Delta SBD}$ and its N148A, N148Y, K131A and R141A variants. The enzymes were incubated in the presence of 10 mM $CaCl_2$ at 40℃ and pH 6.5 for the times indicated. The activity was then assayed in the standard buffer for substrate of insoluble rice starch.

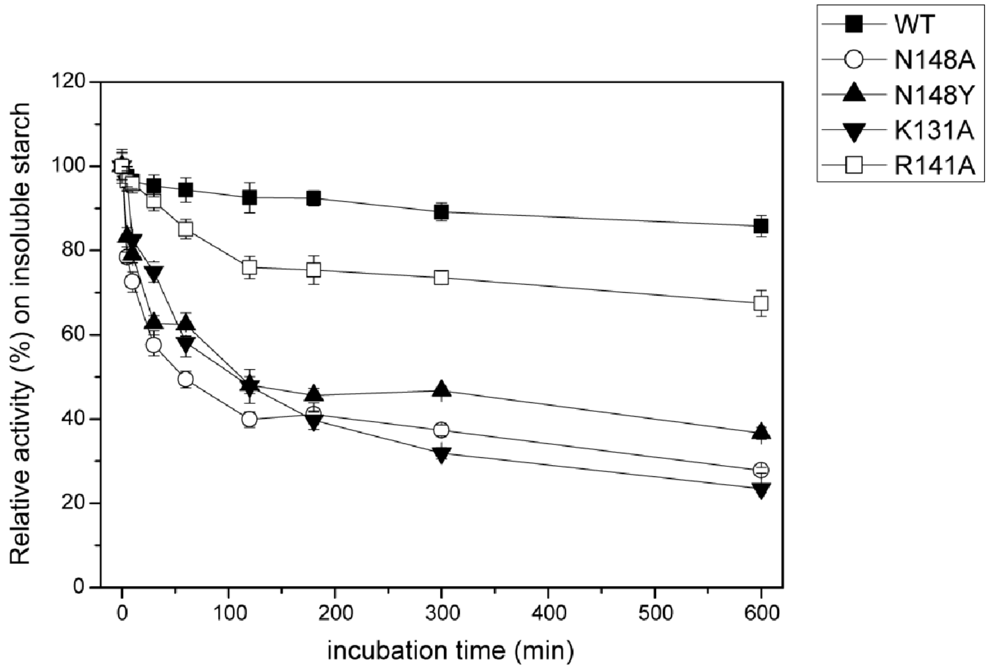

Fig. S11

Structural superposition of AmyA (cyan, pdb accession code 1wza) and GTA complexed with an acarbose-derived pseudo-hexasaccharide (gray and yellow, pdb accession code 4e2o). Surface representation of AmyA is shown. The catalytic residues are colored in red. The structural lid above the active site of AmyA, that is relatively larger in area than that of GTA, is marked by a magenta circle.

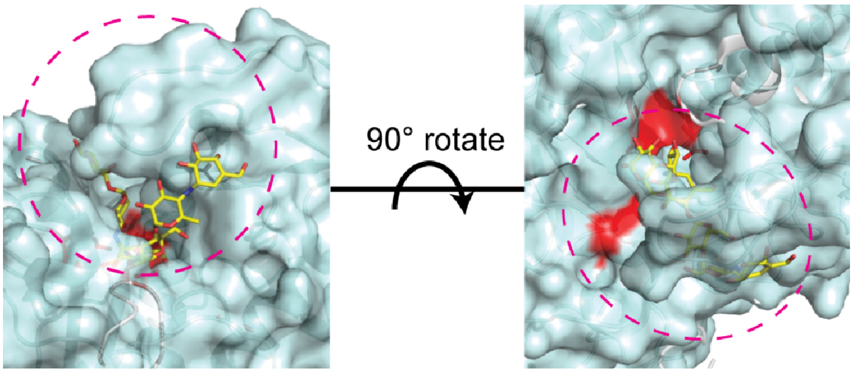

Fig. S12

(a) Far-UV CD and (b) intrinsic fluorescence emission spectra for WT AmyP$_{\Delta SBD}$, its single site mutants (W80A, Y228A, Y252A, and W272A) and multiple site mutants (W80A/Y228A, Y252A/W272A, W80A/Y228A/Y252A, and W80A/Y228A/Y252A/W272A) were recorded.

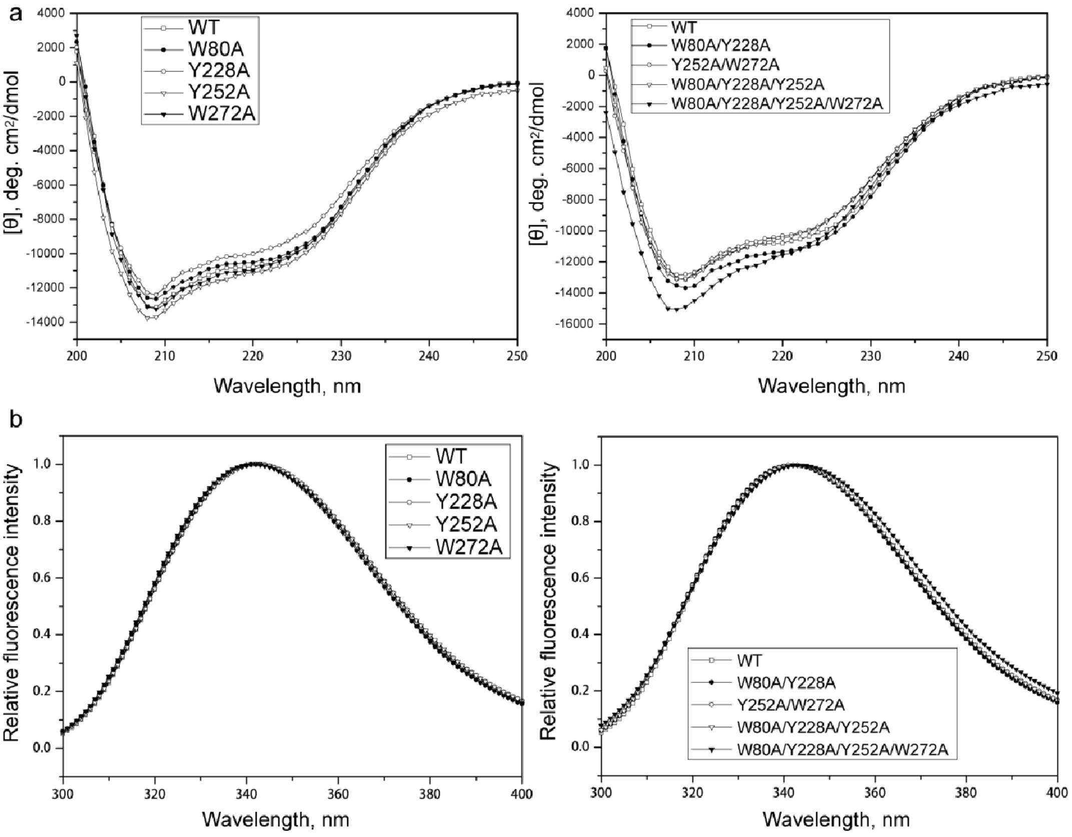

Supplement: Supplementary Information [file srep44067-s1.pdf]
